# Inhibition of MMP-2 Expression with siRNA Increases Baseline Cardiomyocyte Contractility and Protects against Simulated Ischemic Reperfusion Injury

**DOI:** 10.1155/2014/810371

**Published:** 2014-07-24

**Authors:** Han-Bin Lin, Virgilio J. J. Cadete, Bikramjit Sra, Jolanta Sawicka, Zhicheng Chen, Lane K. Bekar, Francisco Cayabyab, Grzegorz Sawicki

**Affiliations:** ^1^Department of Pharmacology, College of Medicine, University of Saskatchewan, 107 Wiggins Road, Saskatoon, SK, Canada S7N 5E5; ^2^Faculté de Pharmacie, Université de Montréal, Montréal, QC, Canada H3T 1J4; ^3^Department of Physiology, University of Saskatchewan, Saskatoon, SK, Canada S7N 5E5; ^4^Department of Clinical Chemistry, Medical University of Wroclaw, 50-556 Wroclaw, Poland

## Abstract

Matrix metalloproteinases (MMPs) significantly contribute to ischemia reperfusion (I/R) injury, namely, by the degradation of contractile proteins. However, due to the experimental models adopted and lack of isoform specificity of MMP inhibitors, the cellular source and identity of the MMP(s) involved in I/R injury remain to be elucidated. Using isolated adult rat cardiomyocytes, subjected to chemically induced I/R-like injury, we show that specific inhibition of MMP-2 expression and activity using MMP-2 siRNA significantly protected cardiomyocyte contractility from I/R-like injury. This was also associated with increased expression of myosin light chains 1 and 2 (MLC1/2) in comparison to scramble siRNA transfection. Moreover, the positive effect of MMP-2 siRNA transfection on cardiomyocyte contractility and MLC1/2 expression levels was also observed under control conditions, suggesting an important additional role for MMP-2 in physiological sarcomeric protein turnover. This study clearly demonstrates that intracellular expression of MMP-2 plays a significant role in sarcomeric protein turnover, such as MLC1 and MLC2, under aerobic (physiological) conditions. In addition, this study identifies intracellular/autocrine, cardiomyocyte-produced MMP-2, rather than paracrine/extracellular, as responsible for the degradation of MLC1/2 and consequent contractile dysfunction in cardiomyocytes subjected to I/R injury.

## 1. Introduction

The pathological role of matrix metalloproteinases (MMPs), including MMP-2, during the development of oxidative stress-mediated cardiac injury and contractile dysfunction has been well described [1–3]. We and others have shown increased activity of MMP-2 in ischemic reperfusion (I/R) injury [4–6], hypoxia-reoxygenation injury [7], and infusion of reactive oxygen species, namely, peroxynitrite (ONOO^−^) [8, 9]. Furthermore, increased intracellular MMP-2 activity [10–13] is associated with degradation of contractile proteins such as troponin I [10], titin [11], myosin light chain 1 (MLC1) [12, 14, 15], and myosin light chain 2 (MLC2) [13]. All of these observations have been made in intact, isolated hearts during a relatively short time-course (minutes) and appear to be independent of changes in collagen content [16, 17], supporting an acute intracellular action of MMP-2.

MMP-2 can be found in most cardiac cell types, including vascular endothelial cells [18], smooth muscle cells [19], fibroblasts [20], and cardiomyocytes [10, 21]. The majority of MMP-2 synthesized is secreted (~60%) acting in a paracrine manner, with the remaining 40% being targeted to the cytosol [22] or mitochondrial associated membranes [23]. Therefore, it is possible that MMP-2 originating in endothelium, smooth muscle cells, or fibroblasts is upregulated in response to oxidative stress and can act in a paracrine manner on cardiomyocytes, contributing to the development of I/R injury and cardiac contractile dysfunction. Hence, the understanding of MMP-2's mechanism of action (paracrine versus autocrine) and determination of the cellular source/targets of MMP-2 are crucial in the development of novel and more selective drug design.

Greater than 20 MMPs have been described to date with all of them showing similarities in substrate specificities and response to known MMP inhibitors [24], limiting the clinical application of these broad spectra drugs. The vast majority of studies, looking at the roles of MMPs in the development of I/R injury, have been performed in whole heart or whole cardiac tissue homogenates, without the ability to discriminate the source between cell types. Moreover, the pharmacological approaches used to modulate MMP activity rely on the use of broad spectra MMP inhibitors (e.g., doxycycline, orthophenathroline) making it difficult to identify isoform specific effects. Inhibition of MMP-2 has been shown to protect isolated cardiomyocyte contractility in response to oxidative stress [21, 25]. Nonetheless, clinical usage of subantimicrobial doses of doxycycline in patients undergoing cardiopulmonary bypass surgery failed to show a protective effect on cardiac function despite the inhibition of MMP-2 [26]. Consequently, despite the wide body of preclinical evidence supporting inhibition of MMP-2 in cardiac pathologies, the failure of clinical translation makes it crucial to determine the physiological and pathological roles of MMP-2 in order to improve therapeutic strategies that target MMPs.

Here we show that specific, autocrine, intracellular action of MMP-2 on cardiomyocytes regulates not only contractile protein turnover under physiological conditions but also the development of I/R-induced cardiac contractile dysfunction,* via* increased degradation of contractile proteins. These observations, made with use of specific siRNA inhibition of MMP-2, provide novel and important knowledge of the role of MMP-2 in I/R injury and indicate potential therapeutic alternatives to the prevention and treatment of I/R injury.

## 2. Materials and Methods

This investigation conforms with the Guide to the Care and Use of Experimental Animals published by the Canadian Council on Animal Care.

### 2.1. Cardiomyocyte Isolation

Male Sprague-Dawley rats (weighing 100–150 g) were anaesthetized with sodium pentobarbital (60 mg/kg i.p.) and hearts were removed. Right ventricular myocytes were used as they provide a consistently higher ratio of live cardiomyocytes to contaminating fibroblasts and endothelial cells in comparison to preparations from the left ventricle [3]. Right ventricular myocytes were obtained by enzymatic dissociation as previously described [27].

### 2.2. Primary Culture of Cardiomyocytes

**Figure 1:**
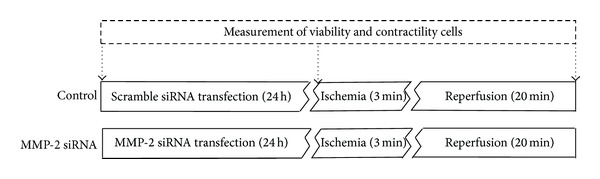
Schematic representation of the perfusion protocol for isolated cardiomyocytes. Scrambled siRNA was used as a control of MMP-2 siRNA. Arrows indicate when cell contractility was measured: (1) before siRNA transfection, (2) before ischemia, and (3) at the end of reperfusion.

Isolated cardiomyocytes were seeded in 35 × 10 mm cell culture plates (Nunc, Roskilde, Denmark) at 2 × 10^5^ cells per plate in DMEM medium (Lonza Walkersville, MD, USA) supplemented with 10% FBS and incubated in a 95% air, 5% CO_2_ incubator at 37°C for 7 hours to stabilize cells. Viability and contractility of isolated cardiomyocytes were assessed after isolation, transfection with siRNA, and at the end of reperfusion (Figure 1).

### 2.3. siRNA Transfection

A mixture containing a pool of 3 target-specific 19–25 nucleotide small interfering RNAs (MMP-2 siRNA) designed to knock down rat MMP-2 gene expression (Santa Cruz Biotechnology, Santa Cruz, CA, USA) was resuspended in RNAse-free water to a final 10 *μ*M concentration in a buffer containing 10 *μ*M Tris-HCl, 20 mM NaCl, and 1 mM EDTA at pH 8.0. This solution was stored at −20°C. As a control, scrambled siRNA (Santa Cruz Biotechnology, Santa Cruz, CA, USA) was used under the same conditions.

Following the initial 7-hour stabilization period in DMEM and 10% FBS, cardiomyocytes were washed with siRNA transfection medium (Santa Cruz Biotechnology, Santa Cruz, CA, USA) and incubated for 24 hours at 37°C in 200 *μ*L of transfection medium containing 0.8 *μ*M of MMP-2 siRNA or scrambled siRNA (Figure 1) according to the manufacturer's protocol. For assessment of transfection efficiency, green fluorescent protein (GFP) was cotransfected with siRNA.

### 2.4. Simulated Ischemia/Reperfusion Protocol

After 24 hours of transfection with siRNA cardiomyocytes were subjected to chemical ischemia, as previously described [28]. Briefly, the transfection medium was removed and replaced with a solution containing 5 mM 2-deoxyglucose to inhibit glycolysis and 4 mM NaCN to inhibit mitochondrial respiration (ischemia). After 1, 3, 5, and 7 minutes of incubation the solution was removed and replaced with culture medium without FBS (reperfusion).

### 2.5. Measurement of Viability and Contractility of Cardiomyocytes

Cardiomyocyte viability and contractility were evaluated at three time points throughout the experimental protocol: 7 hours after cell isolation, after transfection with siRNA (before ischemia), and at the end of reperfusion (Figure 1). The viability of cardiomyocytes was assessed by the trypan blue exclusion test [29–31]. Cardiomyocyte contractility was measured using the IonOptix system and IonWizard 6.0 software (IonOptix, Milton, MA USA). After a stabilization period the chamber containing the cells was perfused with an oxygenated buffer at a constant temperature of 37°C. Cells were continuously paced (stimulated) at 1 Hz and 5 V (IonOptix MyoPacer, Milton, MA, USA). The assessment of cardiomyocyte contractility was made by the measurement of peak shortening, maximal velocity of cell shortening, and maximal velocity of cell relengthening [21] on 8–10 cardiomyocytes per independent experiment, over a 10 min period to give an average measure per sample.

### 2.6. Preparation of Cell Extracts

Cardiomyocytes were collected and stored at −80°C. For biochemical studies, frozen cardiomyocytes were thawed and sonicated on ice twice for 5 seconds in 50 mM Tris-HCl buffer (pH 7.4) containing 3.1 mM sucrose, 1 mM DTT, 10 *μ*g/mL leupeptin, 10 *μ*g/mL soybean trypsin inhibitor, 2 *μ*g/mL aprotinin, and 0.1% Triton X-100. Homogenates were then centrifuged at 10000 g at 4°C for 10 minutes and the supernatant was collected and stored at −80°C until further use. Protein content of the cardiomyocyte extract was measured using the Bradford protein assay (Bio-Rad, Hercules, CA, USA).

### 2.7. Measurement of MMP-2 Activity

Gelatin zymography was performed as previously described [4, 32, 33]. Briefly, homogenates from cardiomyocyte preparations containing 30 *μ*g of protein were applied to 8% polyacrylamide gel copolymerized with 2 mg/mL gelatin. After electrophoresis, gels were rinsed three times for 20 minutes in 2.5% Triton X-100 to remove SDS. The gels were then washed twice in incubation buffer (50 mM Tris-HCl, 5 mM CaCl_2_, 150 mM NaCl, and 0.05% NaN_3_) for 20 minutes at room temperature and incubated in incubation buffer at 37°C for 24 hours. The gels were stained using 0.05% Coomassie Brilliant Blue G-250 in a mixture of methanol : acetic acid : water (2.5 : 1 : 6.5, v : v : v) and destained in aqueous solution of 4% methanol : 8% acetic acid (v : v). Developed gels were scanned with a GS-800 calibrated densitometer and MMP-2 activity was measured using Quantity One 4.6 software (Bio-Rad, Hercules, CA, USA).

### 2.8. Western Blot Analysis

Protein (30 *μ*g) from cardiomyocyte homogenates was separated using 12% SDS-PAGE and transferred to a PVDF membrane (Bio-Rad, Hercules, CA, USA). Myosin light chains 1 and 2 (MLC 1 and MLC2) were identified using mouse monoclonal anti-MLC1 antibody and rabbit polyclonal anti-MLC2 antibody, respectively (Abcam, Cambridge, MA, USA), and MMP-2 was identified using rabbit monoclonal anti-MMP-2 antibody (Abcam, Cambridge, MA, USA). Membranes were developed using Versa Doc 5000 and band densities were measured with Quantity One 4.6 software (Bio-Rad, Hercules, CA, USA). Equal protein loading was additionally verified by measurement of tubulin level with mouse monoclonal antibody (Abcam, Cambridge, MA, USA).

### 2.9. Immunocytochemistry

For immunocytochemistry, cardiomyocytes were seeded on polylysine-coated coverslips and follow the same siRNA transfection protocol. After permeabilization with 0.25% Triton X-100 and fixation, cells were blocked for 1 h with PBS containing 5% bovine serum albumin (BSA, Sigma, St Louis, MO, USA). Proteins of cardiomyocytes were labeled by overnight incubation (at 4°C) with rabbit anti-MMP-2 (Abcam, Cambridge, MA, USA) antibody diluted at 1 : 200 in blocking buffer followed by brief wash (three times; 10 min each) and incubation with Alexa Fluor 555-conjugated goat anti-rabbit secondary antibodies (Invitrogen, Carlsbad, CA, USA) at 1 : 1000 for 1 h. After Hoechst (Sigma, St Louis, MO, USA) staining, the coverslips were mounted on newly cleaned slides using Prolong Gold Antifade Reagent (Invitrogen, Carlsbad, CA, USA) and observed with an LSM700 laser scanning confocal microscope (Carl Zeiss, Oberkochen, Germany). Images were acquired using a Zeiss Plan-Apochromat 63X/1.6 oil objective lens and analyzed with the Zeiss Zen 2009 software (version 5.5 SPI).

### 2.10. Immunoprecipitation

The immunoprecipitation of MMP-2 with MLC1 or MLC2 was initiated by incubating 200 *μ*g of total protein extract with 10 *μ*g mouse anti-MLC1 IgG or 10 *μ*g rabbit anti-MLC2 IgG in a total volume of 500 *μ*L RIPA buffer (150 mM NaCl, 1% IGEPAL CA-630, 0.5% sodium deoxycholate (DOC), 0.1% SDS, 50 mM Tris, pH 8.0, 1 mM PMSF) overnight at 4°C. This buffer was chosen because of its known high stringency to avoid unspecific binding. As a negative control, unrelated IgG was used instead of anti-MLC IgG. Following the initial incubation, 100 *μ*L of slurry of protein A-Sepharose beads was added and the resulting mixture was incubated overnight at 4°C. After overnight incubation the mixture was washed three times with 0.5 mL of RIPA buffer at 4°C and 20 *μ*L of sample buffer was added. Determination of colocalization of MLC1 or MLC2 with MMP-2 was determined by gelatin zymography of the immunoprecipitates.

### 2.11. Statistical Analysis

For contractility measurements, at least three independent experiments (myocyte preparations from different hearts) were run. Each experiment was performed in triplicate (myocytes from the same heart). ANOVA with Kruskal-Wallis post hoc analysis or Student's *t*-tests were used in this study. A *P* < 0.05 indicated statistical significance. Data are presented as the mean ± SEM.

## 3. Results

### 3.1. Cell Contractility and Duration of Ischemia

**Figure 2:**
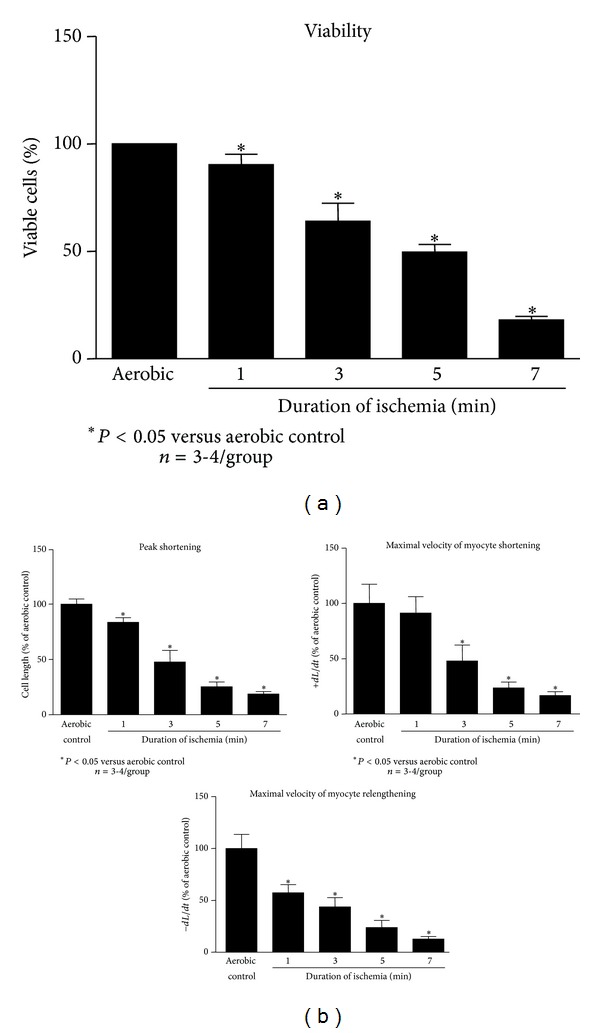
Effect of the duration of ischemia on cardiomyocyte viability (a) and contractility (b). Total number of live cells in aerobic condition is considered as a 100%. *n* = 3-4 heart preparations per group, *P < 0.05 versus aerobic control.

The effect of the duration of ischemia on cardiomyocyte viability and contractility was determined. One minute of ischemia decreased cardiomyocyte viability by approximately 10%, in comparison to aerobic control cells, with longer durations reducing viability further (Figure 2(a)). Cardiomyocyte contractility, namely, peak shortening and maximal velocity of myocyte relengthening, was decreased after 1 minute of ischemia; however maximal velocity of myocyte shortening was unaffected (Figure 2(b)). Three minutes of ischemia reduced contractility by 50% in all three measured parameters. Longer periods of ischemia (5 and 7 min) further reduced contractility to approximately 70–80% of aerobic values (Figure 2(b)). Based on cellular viability and contractility, 3 minutes of ischemia was chosen for further experimentation.

### 3.2. MMP-2 Expression and Activity in Cardiomyocytes Transfected with MMP-2 siRNA

**Figure 3:**
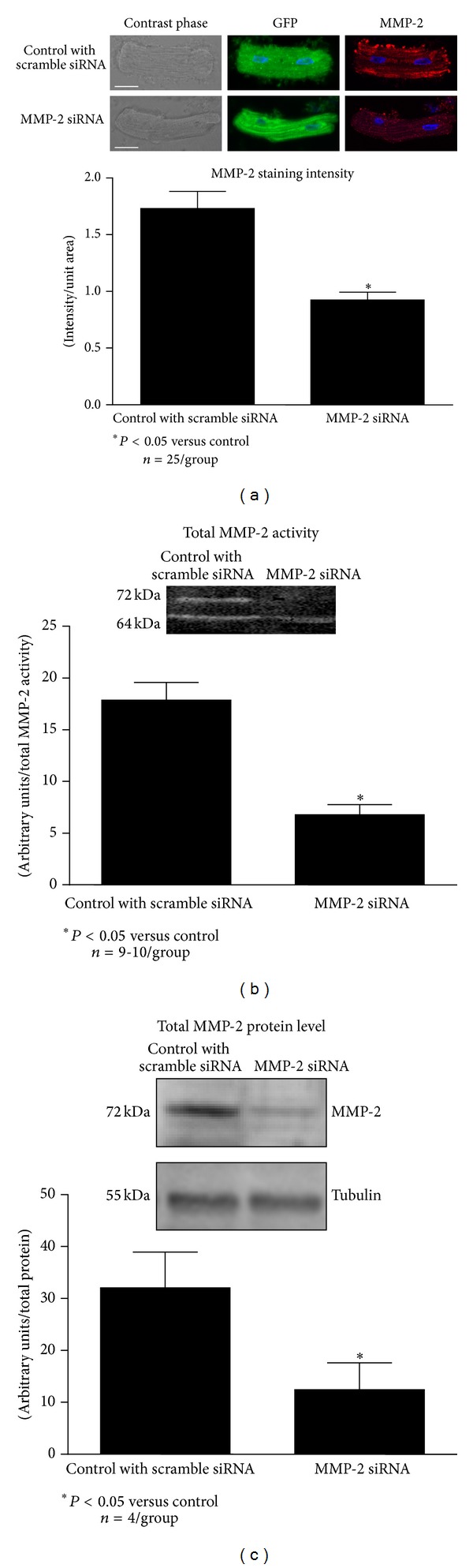
Effect of MMP-2 siRNA transfection on MMP-2 expression in isolated cardiomyocytes. (a) Efficiency of siRNA transfection and MMP-2 protein levels measured by immunocytochemistry. Scale bar, 50 *μ*m. (b) Measurement of MMP-2 gelatinolytic activity by zymography. (c) MMP-2 protein level. (a) *n* = 25 cells from 3 different hearts per group; *n* = 9-10 heart isolates per group for MMP-2 activity (b), in (c) *n* = 4 heart isolates per group, *P < 0.05 versus aerobic control.

MMP-2 siRNA transfection silencing of expression was evaluated by gelatin zymography, immunoblot analysis, and immunocytochemistry using confocal microscopy (Figure 3).

Transfection efficiency, determined by the measurement of GFP-tagged siRNA fluorescence that was cotransfected with either scrambled or MMP-2 siRNA (Figure 3(a), top panel), was approximately 95%. This efficiency of overall transfection was associated with a 50% decrease in the levels of MMP-2, evaluated by immunocytochemistry, in cardiomyocytes transfected with MMP-2 siRNA, in comparison to control cells transfected with scramble siRNA (Figure 3(a)).

Total MMP-2 activity, as determined by gelatin zymography, was reduced by approximately 70% in comparison to control cells transfected with scrambled siRNA (Figure 3(b)). Although the cleaved 64 kDa form of MMP-2 was detected after knocking down MMP-2 mRNA, the pro MMP-2 form (72 kDa) was undetectable (Figure 3(b)). A similar decrease was observed for MMP-2 protein level determined by immunoblotting (Figure 3(c)).

### 3.3. MMP-2 Knockdown Effects on Cardiomyocyte Contractility before and after I/R Injury

**Figure 4:**
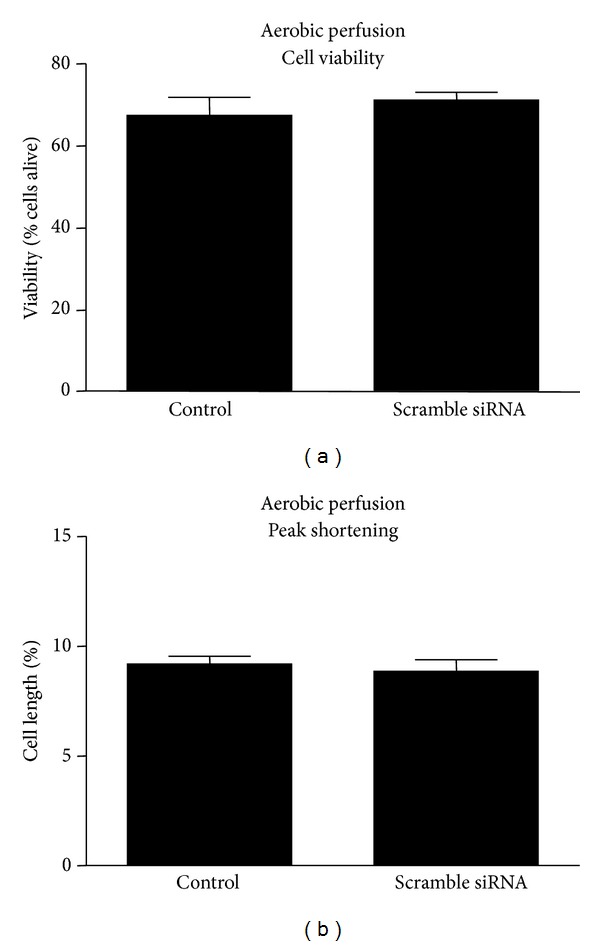
Effect of scrambled siRNA transfection on cardiomyocyte viability (a) and contractility (b). Control cells were transfected with scrambled siRNA. *n* = 4 heart isolations per group.

The effect of siRNA transfection on cell viability and contractility was evaluated using scrambled siRNA to control for possible effects independent of inhibition of MMP-2. Transfection of scrambled siRNA (control) did not impact either cardiomyocyte viability (Figure 4(a)) or contractility (Figure 4(b)).

**Figure 5:**
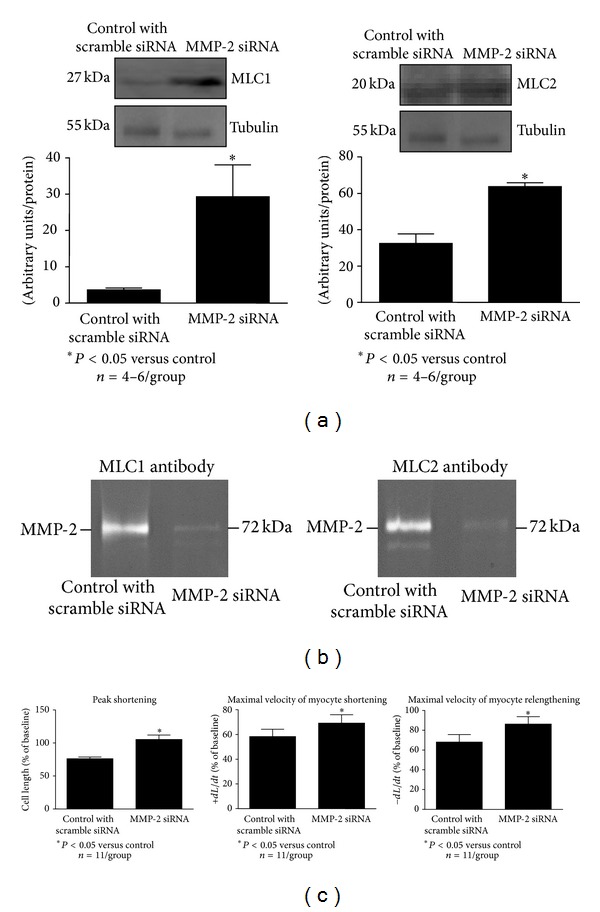
Effect of MMP-2 siRNA transfection on the level of the contractile proteins MLC1 and MLC2 (a), the formation of the complex between MMP-2 and MLC1 or MLC2 (b), and cardiomyocyte contractility (c). As a protein loading control the tubulin level was measured. Control cells were transfected with scrambled siRNA. *n* = 4–6 heart preparation isolations per group. *n* = 11 per group for contractility measurement and *n* = 4–6 for measurement of protein levels. *P < 0.05 versus control.

Transfection of cardiomyocytes with MMP-2 siRNA resulted in an increase in the levels of the sarcomeric proteins myosin light chain 1 and myosin light chain 2 (MLC1 and MLC2, resp.) in comparison to control (Figure 5(a)). This increase in MLC1 and MLC2 was accompanied by a decrease in the formation of the protein complexes MMP-2-MLC1 and MMP-2-MLC2 (Figure 5(b)). A negative control (unrelated IgG to MLC1 or MLC2) did not show formation of complex with MMP-2. These observations at the protein level were associated with an increase in contractile function of aerobically perfused, MMP-2 siRNA transfected cardiomyocytes (before ischemia) in comparison to cells transfected with scrambled siRNA (Figure 5(c)).

**Figure 6:**
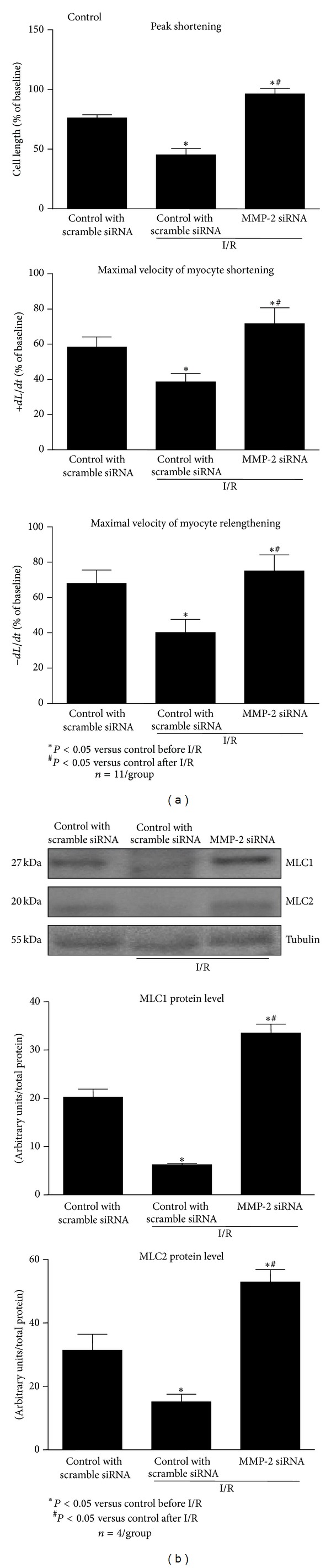
Effect of MMP-2 siRNA transfection on cardiomyocyte contractility (a) and MLC1 and MLC2 in cardiomyocytes subjected to I/R. As a protein loading control the tubulin level was measured. Control cells were transfected with scrambled siRNA. *n* = 11 per group for contractility measurement and *n* = 4 for measurement of protein levels. *P < 0.05 versus control, ^#^ *P* < 0.05 versus I/R.

The contractile function of cardiomyocytes, transfected with scrambled siRNA (control), in response to I/R was significantly decreased, whereas transfection with MMP-2 siRNA fully protected contractile function against I/R (Figure 6(a)). However, it should be noted that the contractility was higher in the MMP-2 knockdown cells than those with scrambled transfection (Figure 6(a)). The protective effects of MMP-2 inhibition by siRNA transfection were associated with increased levels of MLC1 and MLC2 that were 3- and 2-fold higher, respectively, in comparison to control scramble siRNA transfected cells under aerobic conditions (Figure 6(b)).

## 4. Discussion

While the cellular mechanisms of I/R injury are complex and not entirely understood, the degradation of contractile proteins is considered to be a major cause of heart injury [12, 34], with matrix metalloproteinase-2 (MMP-2) playing a significant role in contractile protein degradation [4, 10, 12, 13]. However, degradation of contractile proteins is one from few mechanisms so far described. Metabolic remodeling expressed by ATP depletion, changes in signal transduction, structural remodeling, and oxidative stress are important players in heart failure [35–37].

To the best of our knowledge, this study is the first to demonstrate that, under physiological conditions, MMP-2 regulates MLC1 and MLC2 protein turnover. In response to I/R, intracellular MMP-2 activity increases leading to degradation of these contractile proteins and decreased cardiomyocyte contractility. Inhibition of MMP-2 by siRNA transfection (by reduction in MMP-2 protein levels) protects MLC1, MLC2, and cardiomyocyte contractility from I/R. Moreover, our study demonstrates that MMP-2 acts in an autocrine and intracellular fashion to regulate contractile protein turnover under physiological conditions. Furthermore, our study suggests that, in contrast to broad spectra MMP inhibitors, the use of MMP-2 siRNA to specifically modulate MMP-2 activity can become of clinical relevance in the prevention and treatment of I/R injury and contractile dysfunction associated with loss of contractile proteins.

MMPs are proteolytic enzymes known for their role in maintaining the structural integrity of the extracellular matrix [38]. However, studies over the last decade strongly suggest that MMP-2, in addition to the role in remodeling and degradation of extracellular matrix, is also involved in intracellular degradation of contractile proteins in heart subjected to oxidative stress [3, 39, 40]. This increased degradation reduces sarcomeric integrity resulting in contractile dysfunction of the injured heart [15]. The study of the roles of MMP-2 in the heart has, almost exclusively, focused on pathological conditions. We have previously reported that MMP-2 may be involved in the physiological regulation of contractile proteins, namely, MLC1 and MLC2 [13, 14]. Here we show that the specific inhibition of MMP-2 protein levels and activity, in cardiomyocytes, with siRNA reduces the formation of the protein complex between MMP-2 and MLC1/2, resulting in an increase in MLC1/2 protein levels, in cells cultured under aerobic conditions. Importantly, the increase in MLC1/2 protein levels is associated with increased cardiomyocyte contractility. This observation suggests that inhibition of MMP-2 could be of potential usefulness in the therapeutic management of cardiac pathologies characterized by depressed cardiac function, such as heart failure [41].

Biological studies of specific MMP actions, including MMP-2, have been limited by the lack of selectivity and specificity of commercially available synthetic inhibitors [42] in addition to cytotoxicity [4, 43]. Moreover, genetic knockdown of MMP-2 has failed to provide a ubiquitously adequate model for the study of both the physiological and the pathological roles of MMP-2, since compensatory mechanisms have been observed to occur. For example, in MMP-2 knockout mice with autoimmune encephalomyelitis an increase of MMP-9 expression and activity is observed [44]. To bypass these limitations in modulating MMP-2 activity, use of small interfering RNA (siRNA) appears to be an apt approach. This genetic manipulation causes a transient and significant reduction in MMP-2 protein expression and consequently a reduction in overall enzymatic activity (Figure 3), and likely avoiding induction of potential compensatory mechanisms [45, 46].

Small interfering RNA has proven to be an effective method for reducing gene expression through the use of a small piece of antisense RNA complementary to a gene of interest [47]. In addition, siRNA has been successfully used in preclinical studies focused on cardiac tissue protection [48, 49]. By using MMP-2 siRNA we show that selective inhibition of intracellular MMP-2 protects the levels of myosin light chains 1 and 2 (MLC1/2) and contractility of cardiomyocytes subjected to I/R. Also, we show that the autocrine and intracellular actions of MMP-2 are responsible for contractile dysfunction and MLC1 degradation in I/R injured cardiomyocytes, independent from paracrine and extracellular MMP-2 actions since no other cell types are present. Although we cannot exclude the involvement of other proteolytic enzymes or nonproteolytic pathways in regulating sarcomeric contractility and protein turnover, we believe that the observed effects result mainly from MMP-2 selective silencing, without the enabling of adaptive mechanisms.

In summary, this study provides clear evidence that intracellular MMP-2 plays a crucial role in the heart under both physiological and pathological conditions, namely, at the level of regulation of contraction. The separation of intracellular from extracellular roles of MMP-2 has the potential to provide new directions for studying mechanisms underlying several cardiac pathologies, including heart failure. Furthermore, due to the potential for the use of siRNA therapies in clinical practice, these results can have a significant impact on the development of new approaches for the protection of hearts from reperfusion injury due to myocardial infarction or coronary revascularization.
